# RNA Signature as Potential Diagnostic Marker for Differentiation of Pancreatic Cysts: A Pilot Study

**DOI:** 10.3390/ijms26199680

**Published:** 2025-10-04

**Authors:** Olga Freylikhman, Sabina Seyfedinova, Valeriia Kamalova, Aleksandra Vatian, Alexander Boukhanovsky, Anna Kostareva, Evgenii Solonitsyn, Olga Kalinina

**Affiliations:** 1 Research Laboratory of Molecular Biology and Genetics, Almazov National Medical Research Centre, Saint-Petersburg 197341, Russia; olga1-7@mail.ru; 2 Endoscopy Department, Almazov National Medical Research Centre, Saint-Petersburg 197341, Russia; seysabina000@mail.com (S.S.); lerakamalovalk@gmail.com (V.K.); 3 Research Center “Strong Artificial Intelligence in Industry”, ITMO University, Saint-Petersburg 197341, Russia; alexvatyan@gmail.com (A.V.); avb_mail@mail.ru (A.B.); 4 Institute of Molecular Biology and Genetics, Almazov National Medical Research Centre, Saint-Petersburg 197341, Russia; akostareva@hotmail.com; 5 Department of Faculty Surgery with Clinic, Almazov National Medical Research Centre, Saint-Petersburg 197341, Russia; mail@esolonitsyn.ru; 6 Research Laboratory of Microvesicular Signaling, Department of Laboratory Medicine with Clinic, Institute of Molecular Biology and Genetics, Almazov National Medical Research Centre, Saint-Petersburg 197341, Russia

**Keywords:** endoscopic ultrasound-guided fine-needle aspiration, pancreatic cysts, mucinous cystic neoplasm, intraductal papillary mucinous neoplasm, mucinous cysts, gene expression analysis, RNA diagnostic markers, early detection of pancreatic cancer

## Abstract

The accurate classification of pancreatic cystic lesions remains clinically challenging due to overlapping imaging features and variable malignant potential. Mucinous cystic neoplasms, in particular, require early identification given their premalignant nature. RNA profiling presents a promising alternative to current diagnostic limitations—a molecular lens sharpened by AI-driven pattern recognition. This study aimed to evaluate the diagnostic potential of RNA signatures for differentiating pancreatic cyst subtypes and to clarify their roles in their pathophysiology. The study included 31 patients with pancreatic lesions who underwent endoscopic ultrasound-guided fine-needle aspiration. RNA was extracted from cyst fluid, tissue, and peripheral blood. Expression of 17 target genes was analyzed using qPCR. Gene expression patterns were compared across mucinous cystic neoplasms, serous cystic neoplasms, pseudocysts, adenocarcinoma, and chronic pancreatitis cohorts. Diagnostic accuracy was evaluated via ROC analysis. Mucinous cysts exhibited significant overexpression of *MUC1*, *ITGA2*, *ELOVL6*, and *MUC5AC* genes compared to serous cysts and pseudocysts. *PKM* gene expression correlated with increasing malignant potential. In blood plasma, only *MUC1*, *MUC4*, and *PYGL* were elevated in adenocarcinoma compared to mucinous neoplasms. We identified a distinct RNA signature that can distinguish mucinous cystic neoplasms from benign cystic lesions (serous cysts and pseudocysts), which could be useful for guiding patient management and improving clinical outcomes. Validation in broader cohorts is essential for clinical implementation.

## 1. Introduction

Technical advances in transabdominal imaging have led to an increase in the detection rate of asymptomatic pancreatic cysts, the prevalence of which is now estimated to be approximately 8% [1]. In routine clinical practice, the diagnosis and management of pancreatic cystic lesions are based on a combination of clinical, biochemical, and imaging data. However, this combination remains insufficient for accurate differential diagnoses of various cyst types, which is extremely important for prognoses and oncologic implications. Although pancreatic cystic neoplasms are associated with a high risk of malignant transformation ranging from 10% to 30% depending on the subtype, most of these lesions never become malignant [2]. It has been shown that even in the presence of worrisome features or high-risk stigmata, the majority of resected cysts exhibit only low-grade dysplasia [3]. Therefore, the differentiation of pancreatic cysts in terms of their malignant potential is highly important for the correct patient prognosis, follow-up program, and treatment choice.

Current diagnostic options of cyst fluid analysis include identification of biochemical markers (carcinoembryonic antigen (CEA), glucose and amylase levels), cytological examination, and molecular markers [4]. Thus, mutations in the *GNAS* and *KRAS* genes demonstrate high specificity (98–100%) for distinguishing mucinous neoplasms from benign cystic lesions [5]. However, despite good specificity, these tests often suffer from poor sensitivity, reaching 79% for *GNAS* and *KRAS* mutations, which substantially limits their universal clinical application [6]. Mutations in other genes, including *CDKN2A, PIK3CA, SMAD4*, and *TP53*, are highly specific for high-grade dysplasia or PDAC in mucinous cysts, but their sensitivity ranges from 8% to 80% [7]. The diagnostic utility of biochemical and molecular tests may be further limited by the small volume of aspirated fluid, its high viscosity, and contamination with gastrointestinal tract cells [8]. Considering these limitations, the identification and clinical validation of markers associated with the potential of malignant transformation, particularly those based not on DNA but on transcriptomic data, remain highly relevant needs. This challenge is further complicated by the current lack of transcriptomic data for pancreatic cysts. Molecular profiling of cysts remains limited and is largely extrapolated from pancreatic ductal adenocarcinoma (PDAC), which has been more thoroughly studied [9,10]. Although asymptomatic pancreatic cysts and PDAC have several common signaling pathways and molecular mechanisms of pathogenesis, their comparison is not always justified.

Previously, we demonstrated that the RNA extracted from endoscopic ultrasound-guided fine-needle aspiration (EUS-FNA) samples of various cystic and solid pancreatic lesions exhibits sufficient quality and quantity for a wide range of molecular biological analyses [11]. In a current study, we aimed to evaluate the diagnostic potential of 17 target genes for differentiating pancreatic cyst subtypes (mucinous, serous, and pseudocysts) and to elucidate their roles in pancreatic cyst pathophysiology. Based on the molecular similarities between pancreatic cyst malignant progression and PDAC, we focused on genes previously identified as differentially expressed in PDAC transcriptomic studies. These selections were guided by the AI-informed mining of oncogenic signatures. These genes are involved in epithelial proliferation (cyclin B1 (*CCNB1*)), cell migration and invasion (glycogen phosphorylase L (*PYGL*)), cell cycle regulation (kinesin family member 22 (*KIF22*) and ubiquitin-conjugating enzyme E2 C (*UBE2C*)), cancer metabolism (cyclin dependent kinase 1 (*CDK1*), pyruvate kinase M1/M2 (*PKM*), fatty acid elongase 6 (*ELOVL6*), and N-acyl phosphatidylethanolamine phospholipase D (*NAPEPLD*)), proliferation (bHLH transcription factor (*MYC*)), and cell adhesion (claudin18 (*CLDN18*)). In addition, glypican 1 (*GPC1*)) in combination with membrane-bound mucins (*MUC1*, *MUC4*, *MUC16*), secreting mucins (*MUC5AC*), and two hub genes are upregulated in PDAC (plasminogen activator, urokinase (*PLAU*), and integrin subunit alpha 2 (*ITGA2*)) [12,13,14,15,16,17,18,19,20,21,22,23]. We identified that expression of four genes (*MUC1*, *ITGA2*, *ELOVL6*, and *MUC5AC*) can differentiate mucinous neoplasms (intraductal papillary mucinous neoplasms (IPMNs) and mucinous cystic neoplasms (MCNs)) from pseudocysts and serous cystic neoplasms (SCNs) and that the *PKM* gene is a potential biomarker of the progression of mucinous cystic neoplasms to adenocarcinomas.

## 2. Results

In total, 31 EUS-FNA biopsy samples were included in the study. Corresponding clinical and morphological characteristics of the samples are summarized in Table 1. There were no statistical differences in age, gender distribution, or specimen size between all the groups studied. The distribution of the source of specimen within the pancreas had statistically significant differences.

### 2.1. Gene Expression Profiling in EUS-FNA Samples

**Figure 1:**
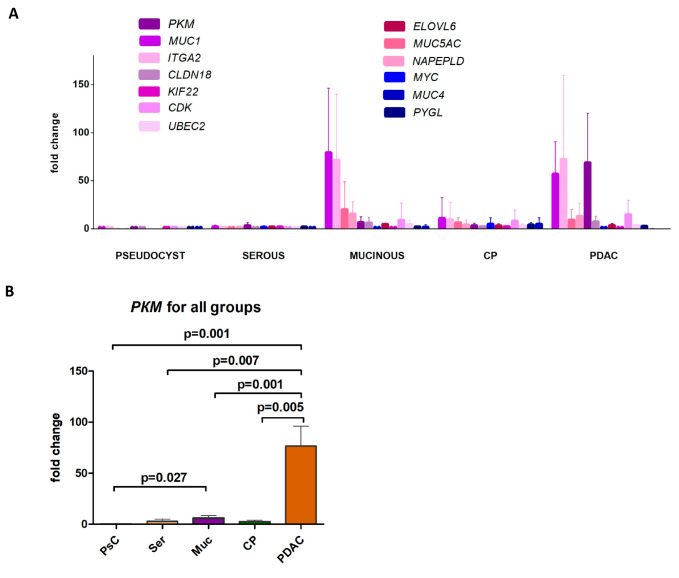
Differential gene expression patterns in EUS-FNA samples from pancreatic cysts (pseudocyst, serous, mucinous), chronic pancreatitis (CP), and adenocarcinoma (PDAC). (**A**) Relative expression patterns of 13 target genes in all cohorts. (**B**) *PKM* expression pattern in all study groups.

In the first step, we compared the gene expression profiles of benign cystic lesions (serous cystic neoplasms and pseudocysts) and malignant tumors (pancreatic adenocarcinomas). Overall, the expression of 13 out of 17 genes was significantly higher in adenocarcinomas compared to benign lesions (Figure 1A). When studying mucinous neoplasms (IPMNs and MCNs), we detected an expression profile similar to that of adenocarcinomas, although the gene expression level was generally higher in the latter. *PKM* was the only gene that showed a statistically significant difference in expression between mucinous cysts and adenocarcinomas (*p* < 0.001). Across all samples studied, its expression level significantly increased with a rise in malignant potential, having the lowest values in pseudocysts, intermediate levels in mucinous neoplasms, and the highest levels in pancreatic adenocarcinomas. The chronic pancreatitis group had low PKM expression, similar to that of benign cystic lesions, without significant differences between it and other cohorts (Figure 1B).

**Figure 2:**
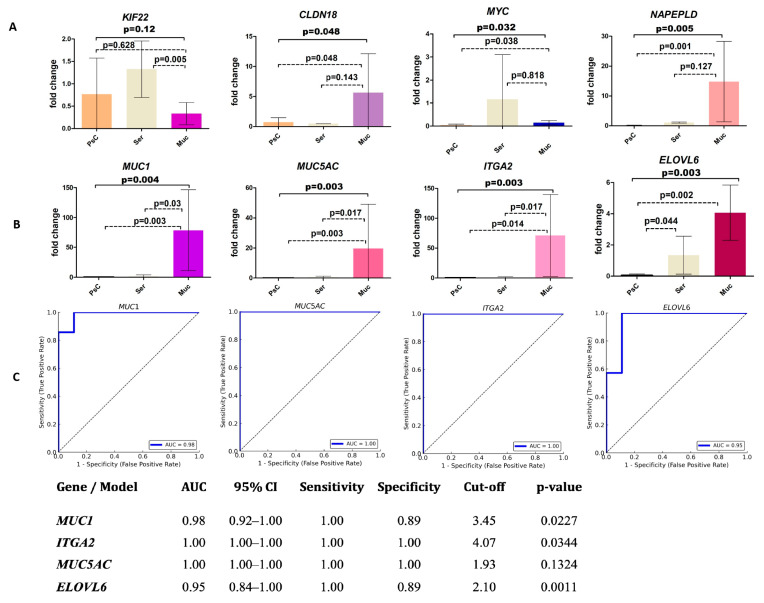
RNA-based molecular markers in cystic fluid for differentiating mucinous neoplasms. (**A**) The relative expression levels of eight target genes in benign cystic lesions (pseudocysts and serous cysts) and mucinous neoplasms. Dashed lines indicate intergroup *p*-values; solid lines represent *p*-values for mucinous cysts versus benign cystic lesions. (**B**) Receiver-operating characteristic (ROC) curves of *MUC1*, *MUC5AC*, *ELOVL6*, and *ITGA2* for differentiating mucinous cysts from benign lesions. (**C**) ROC analysis statistics. CI—confidence interval, ROC—receiver-operating characteristics, AUC—area under the curve. Differences were considered significant at *p* < 0.05.

Additionally, we detected significant differences in the expression levels of five genes between mucinous and serous cysts (*MUC1*, *ITGA2*, *ELOVL6*, *MUC5AC*, and *KIF22*), and of eight genes between mucinous cysts and pseudocysts (*CLDN18*, *NAPEPLD*, *MYC*, *PKM*, *MUC1*, *ITGA2*, *ELOVL6*, and *MUC5AC*) (Figure 2A, Table S1). Overall, four genes (*MUC1*, *MUC5AC*, *ITGA2*, and *ELOVL6*) were differentially expressed in mucinous neoplasms compared to both serous cysts and pseudocysts—a quartet spotlighted not only by biology but also by AI-enhanced analytics. ROC analysis performed for these genes demonstrated their high prognostic potential to differentiate mucinous neoplasms from serous cysts and pseudocysts (Figure 2B,C).

### 2.2. Gene Expression Profiling in Blood Plasma Samples

**Figure 3:**
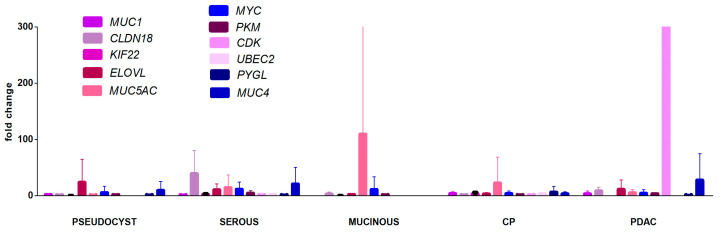
Relative expression patterns of 11 target genes in plasma samples across all study cohorts. CP—chronic pancreatitis, PDAC—pancreatic ductal adenocarcinoma.

To test the diagnostic potential of the markers identified in the biopsy samples, we tested the expression of the abovementioned 17 target genes in the blood plasma samples across all groups. Out of the 17 genes studied, only 11 were detected in the plasma samples (Figure 3, Table S2). Only three out of the 17 genes—*MUC1*, *PYGL*, and *MUC4*—were significantly overexpressed in the adenocarcinoma group compared to the mucinous neoplasm group (*p* = 0.003, *p* = 0.028, and *p* = 0.06, respectively). No other significant differences were found across the groups studied.

## 3. Discussion

The differential diagnosis of pancreatic cysts remains a significant clinical challenge, particularly in light of their heterogeneous biological behavior and varying malignant potential. Several earlier studies demonstrated that aberrant expression of specific genes is associated with cyst malignancy, but there is still no unified molecular approach for their differential diagnosis. At the same time, the combination of molecular markers can supplement classical morphological analysis and increase its accuracy in terms of the identification of potential neoplasms prone to malignant transformation.

To address this clinical challenge, we performed gene expression analysis on various pancreatic cysts as well as pancreatic adenocarcinomas using quantitative PCR. We demonstrated that mucinous cystic neoplasms share a gene expression profile closely aligned with that of pancreatic adenocarcinoma, whereas serous cysts and pseudocysts are characterized by significantly lower expression levels of the abovementioned genes.

Several studies have explored RNA-based markers in pancreatic cystic lesions across different cyst types. The largest of these included 185 cyst fluid FFPE samples investigated using a combined DNA/RNA next-generation sequencing platform (PancreaSeq Genomic Classifier) [24]. This platform evaluated hotspot mutations in 74 genes and assessed the expression of *KRT7*, *KRT20*, *PGK1*, *CHGA*, and *CEACAM5* mRNA, demonstrating high diagnostic accuracy in classifying cystic neoplasms [24]. Early studies have identified a 41-gene signature upregulated during IPMN progression to malignancy by Affymetrix Human Exon microarrays [25], invasion-associated transcripts (claudin 4, CXCR4, S100A4, and mesothelin) in IPMNs by microarray analysis and immunohistochemistry [26], and demonstrated distinct *MUC7* expression patterns between EUS-FNA adenocarcinoma and cystic lesions by RT-qPCR [27]. Despite the absence of a direct overlap in the genes studied, Ziaziaris et al. also demonstrated fundamental differences in the transcriptomic profile of mucinous cysts compared to benign lesions, including genes associated with progression and poor prognosis (*CEACAM6*, *S100A2*, and *MUC16*) [26].

These studies highlight the promise of RNA-based diagnostics for pancreatic cysts. However, identifying molecular markers capable of accurately differentiating cyst types and elucidating the underlying molecular mechanisms, particularly for clinical interpretation, remain challenging [14,23]. Building upon recognized gene targets as diagnostic biomarkers for PDAC, our study compared their expression in various pancreatic cyst subtypes (IPMN, MCN, SCN, and pseudocysts) to investigate their relevance to malignant transformation and progression to pancreatic adenocarcinoma. This creates an AI-assisted bridge between molecular detail and clinical foresight.

The identified overexpression of *MUC1*, *ITGA2*, *ELOVL6*, and *MUC5AC* in mucinous cysts compared to other cyst types points to their potential diagnostic and prognostic relevance. Our findings align with previously published data on mucin-related gene expression—*MUC1*, *MUC4*, and *MUC5AC*—previously identified in association with IPMNs and PDAC. While *MUC1* is normally expressed in epithelial cells, it plays a key role in neoplastic transformation and progression in various epithelial malignancies [27,28,29,30,31]. In the study by Tomishima et al., increased *MUC1* expression was observed in IPMNs, mimicking the expression patterns of ductal adenocarcinoma, and correlated with high-risk features [32]. By contrast, *MUC5AC* is not expressed in normal pancreatic tissue, which implies that it is a potential marker of precancerous lesions such as chronic pancreatitis, pancreatic intraepithelial neoplasms, and mucinous neoplasms, as well as invasive carcinomas. Its neo-associated expression along with *MUC2* and *MUC4* is frequently reported in IPMNs, although no consistent correlation with malignant transformation has been established [22,33]. Similar results were reported by Carrara et al., where the expression of *MUC1* and *MUC5AC* was higher in mucinous cysts and adenocarcinomas, consistent with our data. In their study, *MUC2* was also increased in cystic lesions, which was not confirmed in our cohort, whereas *MUC4* expression was largely absent, while in our samples it demonstrated moderate upregulation [34].

Unlike mucins, the roles of *ITGA2* and *ELOVL6* in pancreatic neoplasm progression are less well defined due to their multifunctionality and ubiquitous tissue expression. Both genes have been reported in association with tumor progression: *ITGA2* contributes to tumor cell migration and invasion and has been identified as a marker of poor prognosis in PDAC, whereas *ELOVL6*—a key regulator of lipogenesis—contributes to the progression of various gastrointestinal cancers [35]. However, their expression in cystic lesions has not been previously reported, and further validation is needed to assess their role in the pathogenesis of mucinous neoplasms.

The similarity in expression profiles between mucinous neoplasms and pancreatic adenocarcinomas supports the hypothesis that mucinous cysts are potentially malignant lesions. Conversely, their expression differences provide a useful diagnostic basis for stratifying cysts by malignant potential. In this regard, we highlighted the *PKM* gene as a potential biomarker for stratifying the malignant potential of pancreatic lesions. Its expression progressively increases from pseudocysts to mucinous neoplasms and PDAC, suggesting its possible involvement in pancreatic carcinogenesis. These findings are consistent with previous reports describing *PKM2* (*PKM* isoform) as a metabolic oncogene promoting glycolysis and tumor cell proliferation in pancreatic cancer and underscore the biological relevance of *PKM* as a marker of early neoplastic transformation [15,36]. Recent pan-cancer transcriptomic analyses have shown that *PKM2* mRNA expression is elevated in the majority of malignancies (over 25 cancer types, e.g., pancreatic cancer, breast cancer, colon cancer, lung adenocarcinoma, etc.) and is associated with worse overall survival, highlighting its conserved role in tumor progression beyond pancreatic cancer [37,38]. The gene expression profile in chronic pancreatitis samples supports the abovementioned trends, showing downregulation of oncogene-associated transcripts (*MUC1*, *MUC4*, *PKM*, and *ITGA2*) relative to adenocarcinomas. Consequently, including the *PKM* gene in a potential molecular diagnostic panel may improve its ability to detect early cancerous transformation in mucinous cysts, which warrants further investigation.

Our findings provide a molecular basis for the expression levels of *MUC1*, *ITGA2*, *ELOVL6*, and *MUC5AC* in the development of a potential molecular diagnostic panel for distinguishing mucinous cystic neoplasms from other pancreatic cyst types. However, the small sample size that was studied remains a major limiting factor, making the clinical relevance of the results obtained after the validation in the larger, independent patient cohorts especially noteworthy.

Despite the identification of potential molecular biomarkers in EUS-FNA specimens from pancreatic tissue, these results are still far from being used for extrapolation compared to less invasive approaches such as blood serum tests. In our study, we were able to detect only the difference between mucinous cysts and PDAC based on *MUC1*, *PYGL*, and *MUC4* plasma gene expression analysis and detected no significantly expressed genes for distinguishing different types of cysts. These results further underscore the diagnostic potential of mucin-related genes in the detection of malignant pancreatic transformations, and also points to the lack of systemic biomarkers of different cyst types, possibly due to the low specific gene expression level and noninvasive character of the benign pathologies.

Our study has several important limitations. First, small sample size remains a major limiting factor. Second, a limited number of studied genes and the target gene approach did not allow us to uncover more potentially relevant genes outside our panel of 17 genes, which is particularly important for serum marker identification. This limitation can be overcome by using broader transcriptomic analyses based on the RNA-sequencing approach in combination with systematic bioinformatics tools that are supercharged by AI pipelines for data-driven discovery.

## 4. Materials and Methods

### 4.1. Study Cohort

The study cohort included 31 patients with various pancreatic lesions who underwent EUS-FNA at the Almazov National Medical Research Centre between October 2019 and July 2022 for the purpose of morphological verification of pancreatic neoplasms. Based on the histological findings, the samples were divided into the following cohorts: mucinous cystic neoplasms (IPMNs and MCNs, n = 7), SCNs (n = 3), pseudocysts (n = 6), pancreatic ductal adenocarcinoma (n = 10), and chronic pancreatitis (n = 7).

The use of the obtained samples for research purposes was performed according to the Helsinki Declaration, and the study protocol was approved by the Almazov National Medical Centre Ethics Committee (approval no. 2101-23, 24 March 2023).

### 4.2. Sample Preparation

The study samples were collected as fresh-frozen cyst fluid and tissue specimen aspirates obtained via EUS-FNA. EUS-FNA samples were processed within one minute after aspiration by transferring the material from the needle into cryovials and immediately placing them into liquid nitrogen containers pre-delivered to the procedure room. The samples were subsequently stored at −80 °C until RNA extraction. In parallel, peripheral blood samples were collected on the day of the procedure using sterile EDTA-Na vacutainers, centrifuged at 3500 rpm for 10 min at room temperature to separate the plasma, and stored at −80 °C until further plasma processing.

### 4.3. Reverse Transcription and Real-Time PCR

Total RNA was extracted from all EUS-FNA biopsy specimens using TRIzol Reagent (Invitrogen, CA, USA), while plasma samples were processed with TRIzol LS (Invitrogen, CA, USA) following the manufacturer’s protocols. RNA was resuspended in 15 μL of nuclease-free H2O (Invitrogen, CA, USA) and stored at −80 °C until further use. Reverse transcription (100 ng of RNA per each sample) was performed using random primers and MMLV RT kit reagents (Evrogen, MSK, Russia).

The artificial intelligence approach using ChatGPT Deep Research 4o was used to search for an appropriate list of genes in connection with various cyst properties and pancreatic adenocarcinoma development (Table S3). Primer design for target gene amplification was performed using the Primer-BLAST platform [39] (Table S4). Quantitative PCR (qPCR) was performed using gene-specific primers for all target genes using a qPCRmix-HS SYBR low ROX reaction mixture (Evrogen, MSK, Russia). Primer design for target gene amplification was performed using the Primer-BLAST platform [39]. To confirm product specificity, a melting curve analysis was performed. PCR reactions for each sample were carried out in technical duplicates on an ABI 7500 (Applied Biosystems, CA, USA) under the following conditions: denaturation at 95 °C for 5 min followed by 40 cycles consisting of 95 °C for 15 s, 60 °C for 30 s, and 70 °C for 30 s.

### 4.4. mRNA Expression Analysis

Relative expression (RQ) values were calculated based on the threshold cycle (Ct) data for each sample using both target and reference gene systems. Amplification efficiency (E) was derived from the slope of the calibration curve using the following formula: E = 10^[−1/slope]. Reactions with amplification efficiency below 95% were excluded from the analysis. Relative expression of target mRNA was calculated based on the ΔCt values between the study and control groups. The 2^−(ΔΔCt)^ algorithm was used for relative quantification.

To select the most suitable reference gene, several commonly used housekeeping genes (such as *GADPH* [40], HPRT [41], and *ACTB* [42]) and genes recommended for PDAC expression studies (EIF2B1 and IPO8 [43]) were evaluated. The *ACTB* gene was ultimately selected based on expression stability, reproducibility, baseline expression level, PCR efficiency, specificity, and performance in the NormFinder statistical algorithm.

### 4.5. Statistical Analysis

Statistical analysis was performed using StatTech v. 4.7.3 (Developer—StatTech LLC, MSK, Russia). Graphs were generated using GraphPad Prism 8.0. A comparison of the expression was made using the Kruskal–Wallis test, and post hoc comparisons were performed using Dunn’s test with the Holm correction. The significance of statistical differences between the two groups was assessed using the independent samples *t*-test or Mann–Whitney U test. Differences were considered significant at *p* < 0.05. Receiver-operating characteristic (ROC) curves were constructed and the area under the curve (AUC) was calculated to determine the diagnostic sensitivity and specificity of the target genes for detecting mucinous cysts.

## 5. Conclusions

This study provides new insights into the molecular landscape of pancreatic cystic lesions through targeted RNA expression profiling of cyst fluid and plasma samples. We identified a distinct gene expression signature that differentiates mucinous cystic neoplasms from other benign cyst types and pancreatic adenocarcinoma. Four genes expressed in cyst fluid—*MUC1*, *ITGA2*, *ELOVL6*, and *MUC5AC*—demonstrated high diagnostic performance in distinguishing mucinous lesions, while *PKM* expression revealed its potential as a marker of malignant transformation. Notably, the integration of tissue- and plasma-based markers (*MUC1*, *PYGL*, and *MUC4*) appeared to improve the discrimination between mucinous neoplasms and PDAC, suggesting the potential feasibility of a combined diagnostic model.

Our data support the concept that mucinous cysts possess molecular features overlapping with pancreatic adenocarcinoma, reinforcing their classification as premalignant lesions. Our findings underscore the potential of RNA-based molecular profiling as a complementary tool for the risk stratification of pancreatic cysts and highlight the importance of future marker validation in larger, independent cohorts for potential clinical implementation.

## Figures and Tables

**Table 1:** Clinical and morphological characteristics of EUS-FNA samples.

Parameters		Study Cohorts					
		IPMN, MCN *n* = 7	SCN *n* = 3	Pseudocyst *n* = 6	Adenocarcinoma *n* = 10	Chronic Pancreatitis *n* = 5	*p*
Patient gender, *n* (%)	Female	3 (42.8)	3 (100)	2 (33.3)	7 (70.0)	1 (20.0)	0.126
	Male	4 (57.2)	0	4 (66.6)	3 (30.0)	4 (80.0)	0.126
Patient age (year), mean [range]		72.4 [56–83]	54.3 [37–70]	51.7 [42–57]	61.1 [44–84]	63.8 [51–77]	0.977
Source of specimen within pancreas, *n* (%)	Head\uncinate	6 (85.7)	0	4 (66.7)	8 (80)	5 (100)	0.020 *
	Body	0	3 (100)	0	0	0	n/a
	Tail	1 (14.3)	0	2 (33.3)	0	0	n/a
	Neck\body\tail	0	0	0	2 (20)	0	n/a
Neoplasm diameter (mm), median [range]		25 [10–38]	40 [24–60]	85 [27–150]	35 [18–39]	-	0.784
Stage of cancer if applicable, *n* (%)	I–II	-	-	-	5 (50.0)	-	n/a
	III–IV	-	-	-	5 (50.0)	-	n/a

*—differences are significant (*p* < 0.05). Abbreviations: IPMN—intraductal papillary mucinous neoplasm, MCN—mucinous cystic neoplasm, SCN—serous cystic neoplasm, n/a—not applicable.

## Data Availability

The raw data supporting the conclusions of this article will be made available by the authors on request.

## Supplementary Materials

The following supporting information can be downloaded at https://www.mdpi.com/article/10.3390/ijms26199680/s1.

## Abbreviations

EUS-FNA	endoscopic ultrasound-guided fine-needle aspiration
CP	chronic pancreatitis
SCN	serous cystic neoplasm
MCN	mucinous cystic neoplasm
IPMN	intraductal papillary mucinous neoplasm
PDAC	pancreatic ductal adenocarcinoma
