# Supplementary material for: RNA Signature as Potential Diagnostic Marker for Differentiation of Pancreatic Cysts: A Pilot Study
Source: Int J Mol Sci. 2025 Oct 4;26(19):9680. doi: 10.3390/ijms26199680 (PMC12525496; doi:10.3390/ijms26199680)
Supplement: Supplementary file 1 [file ijms-26-09680-s001.zip › ijms-3832701-supplementary.pdf]

Supplementary Table S1. Relative expression levels of nine genes in pancreatic cysts EUS-FNA samples.

Genes	FC for mucinous neoplasms, Me [IQR]	FC for other cysts , Me [IQR]	*P value*
CLDN18	4,48 [1,11;6,62]	serous 0,48 [0,48; 0,49]	0,143
		pseudocyst 0,40 [0,27;0,81]	0,048
MUC1	99,41 [12,29; 131,58]	serous 0,31 [0,25;2,32]	0,03
		pseudocyst 0,19 [0,04; 0,31]	0,003
ITGA2	39,94 [17,41; 120,60]	serous 0,06 [0,03;1,13]	0,017
		pseudocyst 0,25 [0,20; 0,57]	0,014
KIF22	0,30 [0,17;0,39]	serous 1,65 [1,12;1,69]	0,005
		pseudocyst 0,47 [0,07;1,95]	0,628
ELOVL6	4,55 [2,35; 5,36]	serous 1,35 [0,74;1,96]	0,044
		pseudocyst 0,03 [0,01;0,05]	0,002
MUC5AC	8,45 [2,21; 20,34]	serous 0,10 [0,07;0,77]	0,017
		pseudocyst 0,02 [0,01;0,09]	0,003
NAPEPLD	10,59 [6,30; 19,16]	serous 1,06 [0,93;1,28]	0,127
		pseudocyst 0,04 [0,02;0,12]	0,001
MYC	0,11 [0,08; 0,21]	serous 0,07 [0,06; 1,74]	0,818
		pseudocyst 0,02 [0,01;0,02]	0,038
PKM	2,14 [1,01; 11,26]	serous 2,88 [0,35;6,91]	0,463
		pseudocyst 0,03 [0,02;1,01]	0,044

FC - fold change; *P* values were calculated using Student's t test. Statistical significance threshold: $p < 0.05$.

Supplementary Table S2. Relative expression levels of 17 target genes in EUS-FNA samples and plasma samples.

Genes	EUS-FNA samples		*P value*	Plasma samples		*P value*
	FC for mucinous neoplasms, Me [IQR]	FC for PDAC, Me [IQR]		FC for mucinous neoplasms, Me [IQR]	FC for PDAC, Me [IQR]	

PYGL	1,51 [0,05-3,87]	1,84 [0,21-5,72]	0,646	0,07 [0,03-0,13]	0,38 [0,04-0,74]	0,028*
MUC1	78,63 [3,45-40,41]	61,16 [0,15-104,69]	0,789	0,01 [0,01-0,21]	0,96 [0,22-16,57]	0,003*
MUC4	0,04 [0,02-0,35]	0,05 [0,02-0,35]	0,547	0,06 [0,03-0,48]	10,33 [2,62-123,86]	0,006*
PKM	6,02 [0,04-15,45]	71,96 [7,82-162,08]	0,006	0,94 [0,42-2,41]	2,36 [0,02-6,67]	0,207
CLDN18	4,48 [1,11; 6,62]	4,86 [1,38; 13,01]	0,791	0,04 [0,04-3,73]	7,25 [4,22 – 11,31]	0,275
GPC1	0,13 [0,10; 0,15]	0,16 [0,10; 0,33]	0,266	0,11 [0,03-0,65]	0,64 [0,44-3,24]	0,196
CDK1	0,36 [0,18; 0,75]	7,09 [4,52; 20,13]	0,079	0,06 [0,003-0,10]	0,77 [0,04-522,39]	0,357
CCNB1	0,08 [0,08; 0,17]	0,10 [0,08; 0,30]	0,710	0,17 [0,14-14,50]	0 [n.d.]	N.D.
UBE2C	4,02 [0,53-12,43]	2,99 [0,04-5,51]	0,596	2,32 [0,53-12,43]	0 [n.d.]	N.D.
PLAU	0,10 [0,08; 0,22]	0,16 [0,12; 0,24]	0,266	0,38 [0,07-0,78]	1,60 [0,57-10,66]	0,245
MYC	0,11 [0,08; 0,21]	0,11 [0,10; 0,32]	0,709	0,07 [0,04-0,66]	0,64 [0,44-3,24]	0,242
ELOVL6	4,07 [2,1-6,39]	3,00 [0,1-7,49]	0,377	1,62 [0,08-2,58]	4,63 [0,48-2,58]	0,143
NAPEPLD	14,81 [1,72-40,41]	13,07 [0,05-44,55]	0,813	N.D.	N.D.	N.D.
KIF22	0,30 [0,17; 0,39]	0,23 [0,13; 0,34]	0,832	0,04 [0,03-0,21]	0 [n.d.]	N.D.
ITGA2	39,94 [17,41; 120,60]	38,06 [8,72; 114,75]	0,958	N.D.	N.D.	N.D.
MUC5AC	8,45 [2,21; 20,34]	5,66 [2,93; 6,87]	0,560	0,28 [0,01-2,84]	1,31 [0,38-8,82]	0,253
MUC16	0,08 [0,01; 0,15]	0,16 [0,06; 0,30]	0,366	N.D.	N.D.	N.D.

EUS-FNA - endoscopic ultrasound-guided fine-needle aspiration, PDAC – pancreatic ductal adenocarcinoma. FC - fold change; *P* values were calculated using Student's t test. Statistical significance threshold: $p < 0.05$; N.D. - non detected

Supplementary **Table S3.** Functional annotation and differential expression patterns of the studied genes

Gene name, accession number	Summary of coding protein functions according to NCBI database annotation	Upregulation or downregulation in described pathological conditions, [references]
CLDN18 NM_001002026.3	A member of the claudin family, integral membrane proteins and components of tight junction strands.	Upregulation [12]
GPC1 NM_002081.3	Glypican 1, this Cell surface heparan sulfate proteoglycans are composed of a membrane-associated protein core substituted with a variable number of heparan sulfate chains.	Upregulation [13]
CDK1 NM_001786.5	Cyclin dependent kinase 1. This protein is a member of the Ser/Thr protein kinase family. This protein is a catalytic subunit of the highly conserved protein kinase complex known as M-phase promoting factor (MPF), which is essential for G2/M phase transitions of eukaryotic cell cycle.	Upregulation [14]
CCNB1 NM_031966.4	Cyclin B1. This protein is a regulatory protein involved in mitosis.	Upregulation [14]
UBE2C NM_007019.	A member of the E2 ubiquitin-conjugating enzyme family. The modification of proteins with ubiquitin is an important cellular mechanism for targeting abnormal or short-lived proteins for degradation.	Upregulation [14]
PLAU NM_002658.6	Plasminogen activator, urokinase, a secreted serine protease that converts plasminogen to plasmin. The encoded preproprotein is proteolytically processed to generate A and B polypeptide chains.	Upregulation [15]
MYC NM_002467.6	MYC proto-oncogene, bHLH transcription factor and encodes a nuclear phosphoprotein that plays a role in cell cycle progression, apoptosis and cellular transformation.	Downregulation [15]
PKM NM_002654.6	Pyruvate kinase M1/M2, a protein involved in glycolysis that catalyzes the transfer of a phosphoryl group from phosphoenolpyruvate to ADP, generating ATP and pyruvate.	Upregulation [15]
ELOVL6 NM_024090.3	ELOVL fatty acid elongase 6, which uses malonyl-CoA as a 2-carbon donor in the first and rate-limiting step of fatty acid elongation.	Upregulation [16]
NAPEPLD NM_001122838.3	N-acyl phosphatidylethanolamine phospholipase D, is an enzyme that catalyzes the release of N-acylethanolamine (NAE) from N-acyl-phosphatidylethanolamine	Upregulation [16,17]

	(NAPE) in the second step of the biosynthesis of N-acylethanolamine.	
KIF22 NM_007317.3	Kinesin family member 22, is a member of the kinesin-like protein family. The family members are microtubule-dependent molecular motors that transport organelles within cells and move chromosomes during cell division.	Downregulation [18,19]
PYGL *NM_002863.5*	Glycogen phosphorylase L, a homodimer protein that catalyzes the cleavage of alpha-1,4-glucosidic bonds to release glucose-1-phosphate from liver glycogen stores.	Upregulation [18,20]
ITGA2 NM_002203.4	Integrin subunit alpha 2 of a transmembrane receptor for collagens and related proteins, which mediates the adhesion of platelets and other cell types to the extracellular matrix.	Upregulation [21]
MUC1 NM_001204291.1	Mucin 1, cell surface associated, a membrane-bound protein that is a member of the mucin family that plays an essential role in forming protective mucous barriers on epithelial surfaces. These proteins also play a role in intracellular signaling.	Upregulation [14]
MUC5AC NM_001304359.2	Mucin 5AC, oligomeric mucus/gel-forming, predicted to be an extracellular matrix structural constituent. Predicted to act upstream of or within maintenance of lens transparency.	Upregulation [22]
MUC4 NM_018406.7	Mucin 4, cell surface associated, The major constituents of mucus, the viscous secretion that covers epithelial surfaces. These glycoproteins play important roles in the protection of the epithelial cells and have been implicated in epithelial renewal and differentiation.	Upregulation [23]
MUC16 NM_001414686.1	Mucin 16, cell surface associated, is a membrane-tethered mucin that contains an extracellular domain at its amino terminus, a large tandem repeat domain, and a transmembrane domain with a short cytoplasmic domain. This protein is thought to play a role in forming a barrier, protecting epithelial cells from pathogens.	Upregulation [23]

Supplementary Table S4. Gene-specific primer sequences for target and reference genes.

Target gene	Forward primer, 5′- 3′	Reverse primer, 5′- 3′	References
CLDN18	CGCTCCTGTGTCCGAGAG	GCAGCATGGCTGGCA	This study
GPC1	GGACCCGGCCAGCAAG	GCAGGTGCTCACCCGAGAT	This study
CDK1	AGCCGGGATCTACCATACCC	CAACTCCATAGGTACCTTCTCCA	This study
CCNB1	AACATCTGGATGTGCCCCTG	CTGACTGCTTGCTCTTCCTCA	This study
UBEC2C	GGGGTCCGGTGGGCAA	CCTCAGGTCTTCATATACTGTTCCA	This study
PLAU	CCAGGGTCCACCTGTCC	AGTTCATTGCTGCCTTTGGA	This study
MYC	CTCCGTCCTCGGATTCTCT	CTCATCTTCTTGTTCCTCCTCAGA	This study
PKM	TCTTCGTCTTTGCAGCGTAG	ATGTGCTCCAGGAATGTGTC	This study
ELOVL6	AGACAGGAGAACACTCGAAATC	ACAGGAAAGATTTCTTCCAGTTTT	This study
NAPEPLD	TTTGAATTTTCGCACCTCGC	CATGTCCTTTGGTGAAGAAATGT	This study
KIF22	GCGATCTCAGGAGCTGGT	CGCTGTTCCATCCACAAATG	This study
PYGL	GGAATTTTACATGGGCCGAAC	TTCTATATCCAATCCAAGCTGGT	This study
ITGA2	ACAAGTGGGATTCAGTGCAG	GCAGCCACAGAGTAACCTAA	This study
MUC1	ATCTCATTGCCTTGGCTGTC	GGGGTACTCGCTCATAGGAT	This study
MUC5AC	CGGGAACCTACTACTCGAACT	CTTGGGGGCAAGTGGTCATAG	This study
MUC4	TGGAGGAGGGTCCCCTGGGT	CGCCGGCTTGAAGAGTGGGG	This study
MUC16	GCTGCCCACAGGAGGAAT	TATCAGGGCACAGGTCTTCT	This study
EIF2B1	GGAAGGAGTTGTTGAAAACGGAG	GGAAGGGGCAGTGTAGTCG	[39]
IPO8	GCAGTAGATGCTGTGGTGGG	GGGTTGTCAAGGTCAAGTGGAG	[39]
ACTB	AGAAAATCTGGCACCACACC	TAGCACAGCCTGGATAGCAA	[38]
GADPH	AATGAAGGGGTCATTGATGG	AAGGTGAAGGTCGGAGTCAA	[36]
HPRT1	ATGAACCAGGTTATGACCTTGAT	CCTGTTGACTGGTCATTACAATA	[37]
